# Necrosis score as a prognostic factor in stage I–III colorectal cancer: a retrospective multicenter study

**DOI:** 10.1007/s12672-023-00655-w

**Published:** 2023-05-08

**Authors:** Huifen Ye, Yiting Wang, Su Yao, Zaiyi Liu, Changhong Liang, Yaxi Zhu, Yanfen Cui, Ke Zhao

**Affiliations:** 1 Department of Radiology, Guangdong Provincial People’s Hospital (Guangdong Academy of Medical Sciences), Southern Medical University, Guangzhou, China; 2 The Second School of Clinical Medicine, Southern Medical University, Guangzhou, China; 3 Guangdong Provincial Key Laboratory of Artificial Intelligence in Medical Image Analysis and Application, 106 Zhongshan Er Road, Guangzhou, 510080 China; 4 Department of Pathology, The Sixth Affiliated Hospital of Sun Yat-Sen University, 26 Yuan Cun 2 Cross Road, TianHe District, Guangzhou, 510655 China; 5 Department of Pathology, Guangdong Provincial People’s Hospital (Guangdong Academy of Medical Sciences), Southern Medical University, Guangzhou, China; 6 Department of Radiology, Shanxi Cancer Hospital, Shanxi Medical University, No.3, Xinjie West Alley, Taiyuan, 030013 China; 7 Guangdong Cardiovascular Institute, Guangdong Provincial People’s Hospital, Guangdong Academy of Medical Sciences, Guangzhou, China

**Keywords:** Necrosis score, Colorectal cancer, Whole-slide images, Overall survival, Disease free survival

## Abstract

**Background:**

Tumor necrosis results from failure to meet the requirement for rapid proliferation of tumor, related to unfavorable prognosis in colorectal cancer (CRC). However, previous studies used traditional microscopes to evaluate necrosis on slides, lacking a simultaneous phase and panoramic view for assessment. Therefore, we proposed a whole-slide images (WSIs)-based method to develop a necrosis score and validated its prognostic value in multicenter cohorts.

**Methods:**

Necrosis score was defined as the proportion of necrosis in the tumor area, semi-quantitatively classified into 3-level score groups by the cut-off of 10% and 30% on HE-stained WSIs. 768 patients from two centers were enrolled in this study, divided into a discovery (N = 445) and a validation (N = 323) cohort. The prognostic value of necrosis score was evaluated by Kaplan–Meier curves and the Cox model.

**Result:**

Necrosis score was associated with overall survival, with hazard ratio for high vs. low in discovery and validation cohorts being 2.62 (95% confidence interval 1.59–4.32) and 2.51 (1.39–4.52), respectively. The 3-year disease free survival rates of necrosis-low, middle, and high were 83.6%, 80.2%, and 59.8% in discovery cohort, and 86.5%, 84.2%, and 66.5% in validation cohort. In necrosis middle plus high subgroup, there was a trend but no significant difference in overall survival between surgery alone and adjuvant chemotherapy group in stage II CRC (*P* = .075).

**Conclusion:**

As a stable prognostic factor, high-level necrosis evaluated by the proposed method on WSIs was associated with unfavorable outcomes. Additionally, adjuvant chemotherapy provide survival benefits for patients with high necrosis in stage II CRC.

**Supplementary Information:**

The online version contains supplementary material available at 10.1007/s12672-023-00655-w.

## Introduction

After years of exploration and practice, substantial advances have been made in early diagnosis, treatment, and prognosis of colorectal cancer (CRC). However, it remains a disease with high morbidity and mortality [1, 2]. The increasing tendency in the number of patients with CRC motivates continued research on prognostic factors. Presently, numerous clinicopathological factors for predicting prognosis enable to identify and screen the CRC patients at high risk, but there is still ill-defined evidence [3, 4]. The characteristic of a malignant tumor is that the cell has unlimited proliferation ability. Tumor necrosis results from the inability to meet the requirement for the rapid proliferation of tumor cells [5, 6]. The extent of necrosis reveals the degree of hypoxia in the tumor [7]. In addition, increased cellular hypoxia in solid tumors also affects metastatic potential and prognosis [8]. At present, tumor necrosis assessment has been successfully applied in renal cancer [9, 10], lung cancer [11], breast cancer [12], upper tract urothelial carcinoma [13], and CRC [14]. Therefore, necrosis as a potential marker of prognostic biomarker deserves exploration and validation. However, most current studies were single-center studies, and biases in patient selection inevitably exist. Because an instructive prognostic indicator could refine and update tumor-node-metastasis (TNM) stage and have a straight impact on the cancer care, multicenter validation of its prognostic value is warranted before applying the necrosis score to clinical practice.

On the other hand, with the progress of treatment, the prognosis of CRC has improved to some extent [15]. Treatment options for CRC still heavily relies on TNM stage [16]. Undoubtedly, TNM is the most important pathological classification in all international CRC guidelines. Nevertheless, clinical outcomes of patients with the same stage could be heterogeneous, due to the differences in clinical and molecular phenotypes, patterns of genetic damage, and host immune responses [17]. Currently, one of the most critical clinically relevant requirements is lack of sufficient predictive biomarkers that can identify patients at high risk, particularly in stage II CRC patients. These high-risk patients may benefit from adjuvant chemotherapy [15]. Therefore, exploring biomarkers such as necrosis to identify high risk CRC patients in stage II will allow use of adjuvant chemotherapy in a select subgroup of high risk patients to improve their prognosis.

There are two aims involved for this study. First, we proposed a necrosis score using HE-stained whole-slide images (WSIs) and validated its prognostic value in two CRC cohorts. Predictive competence of necrosis score in curative effect of adjuvant chemotherapy in stage II CRC was further investigated.

## Materials and methods

### Patient cohort

The inclusion criteria were patients with histologically confirmed stage I–III CRC who underwent surgical resection with curative intent and had paraffin-embedded tumor samples available. The discovery cohort consists of CRC patients from Shanxi Cancer Hospital and the validation cohort from The Sixth Affiliated Hospital of Sun Yat-sen University (Jan 2014 to Dec 2014). This study was approved by the Research Ethics Committees of the respective hospitals, with the need for informed consent waived for this retrospective study. Samples were excluded if the patients had other tumors, received neo-adjuvant therapy (radiotherapy, chemotherapy), follow-up information missing, death within 30 days of surgery, or HE-stained WSIs unavailable. The sample size for the analysis was based on pathological evaluation availability.

Clinicopathological characteristics information was collected from medical records, including age, sex, tumor location (colon or rectum), grade, TNM stage, carcinoembryonic antigen (CEA) level (cutoff = 5 ng/mL, normal = 0–5 ng/mL), microsatellite instability (MSI) status, and post-surgery treatment (surgery alone or adjuvant chemotherapy, selected by clinicians according to whether there were high-risk factors). Supplementary Table 1 shows the distributions of post-surgery treatment of stage II CRC patients. TNM stage was performed according to the Union for International Cancer Control (UICC) guideline [18]. MSI status was determined by four microsatellite markers (MLH1, MSH2, MSH6, and PMS2) if available. Clinical follow-up information was retrieved from the patient's electronic medical record and telephone communication. The prespecified primary endpoint was overall survival (OS), referring to the time from diagnosis to death for any reason. Disease-free survival (DFS), the secondary endpoint of interest, was defined as the date of the first event of cancer recurrence was used. Tumor recurrence is defined as the local recurrence, first distal metastasis or without events but with death. If no recurrence occurred, DFS was calculated as the period until the date of last follow-up.

### Evaluation of necrosis score

The pathologist sampled representative areas in tumor center and edge. Surgical specimens were formalin-fixed, paraffin-embedded, cut into 4 μm slices, and then stained with HE. These HE-stained slides were all scanned by digital whole-slide scanning systems (NanoZoomer S60 C13210-01, Hamamatsu, Japan; SQS-120P, Shengqiang, China) at 40 × magnification (resolution: 0.20–0.23 μm/pixel). Evaluators, blinded to the patient's clinical information and outcome, examined all available WSIs of the primary tumor. Simultaneous opening of all slices of the same patient on HE-stained WSIs. The existence of necrosis was carefully recognized, which is characterized by agglomerated cells forming condensates containing nuclear and cytoplasmic fragments [19]. Similar to previously criteria [14], the extent of necrosis was semi-quantitatively assessed at low magnification (× 5) and recorded as either low (absent and < 10% of the tumor area), middle (10–30% of the tumor area), or high (≥ 30% of the tumor area) based on all available WSIs. To test the interrater agreement, a random subset of 100 cases from the study population was selected, and necrosis score were independently scored by two authors (HFY and KZ). The rest of WSIs were assessed by one author (HFY).

### Statistical analysis

Clinicopathological characteristics were compared by Student t-test for a continuous variable or Chi-square test for a category variable. Cohen’s Kappa was calculated as a statistical measure of the interrater agreement. Kaplan–Meier curves were plotted to determine the difference in survival rates among different groups, and log-rank tests were used to calculate *P* values. The multivariate Cox proportional hazard model was used to examine the associations between factors with OS and DFS. For multivariate analysis, univariate variables with *P* < 0.05 were selected. Hazard ratio (HR) with 95% confidence interval (CI) was calculated by using the Cox model. The discrimination performance of factors was assessed using Harrell's C-statistics (C-index) with 95% CI. Patients with missing data, such as available HE-stained WSIs and follow-up information were excluded, and the imputation method was not used in the analysis. All statistical analyses were conducted in an R environment (version 4.0.3), with statistical significance set at 0.05.

## Results

### Patients

**Fig. 1:**
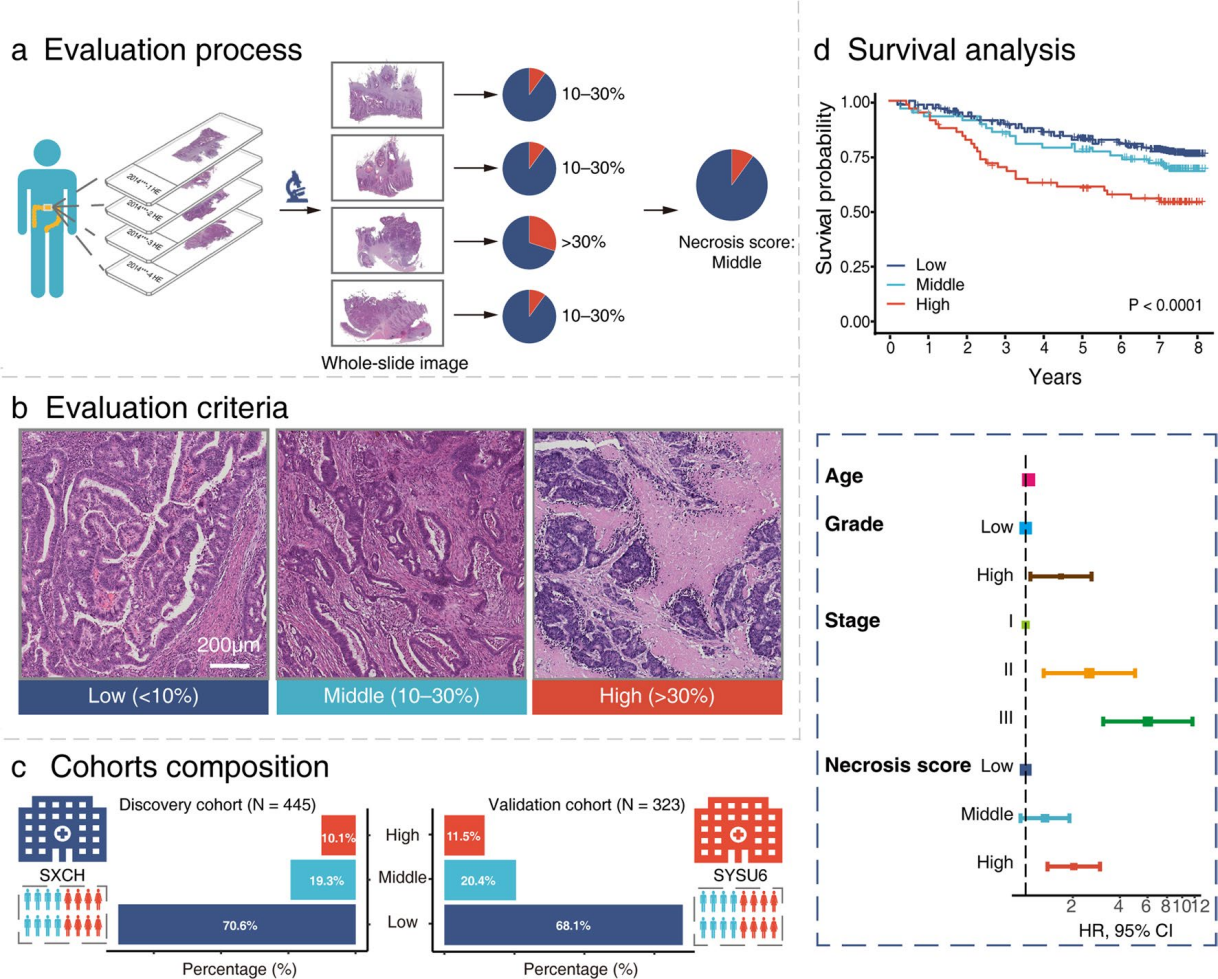
Study workflow. **a** Evaluators examined all available HE-stained WSIs of the primary tumor and scored each of them according to the evaluation criteria, and finally obtained the final necrosis score. **b** The extent of tumor necrosis was semi-quantitatively assessed at low magnification (× 5) and recorded as either low (absent and < 10% of the tumor area), middle (10% − 30% of the tumor area), or high (≥ 30% of the tumor area). **c** The discovery cohort (N = 445) consists of CRC patients from SXCH, and the validation cohort (N = 323) from SYSU6. **d** Kaplan–Meier plots for all patients according to necrosis score and multivariate analysis for OS. *HE* hematoxylin and eosin, *WSIs* whole-slide images, *CRC* colorectal cancer, *SXCH* Shanxi Cancer Hospital, *SYSU6* The Sixth Affiliated Hospital of Sun Yat-sen University

The overall workflow of our study is illustrated in Fig. 1. Tumor samples were collected from 768 patients from two centers with UICC TNM stage I–III CRC. Among them, 445 patients (232 males and 213 females; mean age 59.2 ± 11.9 years) formed the discovery cohort, with a median follow-up time of 7.67 (interquartile range [IQR], 7.33–7.92) years; 323 patients (180 males and 143 females; mean age 60.1 ± 13.0 years) formed the validation cohort, with a median follow-up time of 7.17 (IQR, 6.96–7.43) years. A comparison of clinicopathologic characteristics between the two cohorts is displayed in Table 1.

**Table 1:** The distributions of demographic and clinicopathologic characteristics of colorectal cancer patients in the two cohorts

	Discovery cohort	Validation cohort	*P*
Age (year, mean ± SD)	59.2 ± 11.9	60.1 ± 13.0	0.283^*^
Sex			0.362^#^
Male Female	232 (52.1%) 213 (47.9%)	180 (55.7%) 143 (44.3%)	
TNM stage			0.568^#^
I	72 (16.2%)	61 (18.9%)	
II	193 (43.4%)	140 (43.3%)	
III	180 (40.4%)	122 (37.8%)	
Location			0.002^#^
Colon	192 (43.1%)	176 (54.5%)	
Rectum	253 (56.9%)	147 (45.5%)	
CEA level			0.061^#^
Normal	341 (76.6%)	228 (70.6%)	
Abnormal NA	93 (20.9%) 11 (2.5%)	87 (26.9%) 8 (2.5%)	
Grade			0.480^#^
Low	388 (87.2%)	288 (89.2%)	
High NA	36 (8.1%) 21 (4.7%)	21 (6.5%) 14 (4.3%)	
Necrosis score			0.743^#^
Low Middle High	314 (70.6%) 86 (19.3%) 45 (10.1%)	220 (68.1%) 66 (20.4%) 37 (11.5%)	

*P*-value was performed by t-test or χ^2^ test where appropriate. (^*^t-test; ^#^Chi-square test)

*SD* standard deviation, *TNM* tumor-node-metastasis, *CEA* carcinoembryonic antigen

In the discovery cohort, 314 (70.6%) patients were grouped as necrosis-low, and 86 (19.3%) as middle, and 45 (10.1%) as high. 220 (68.1%) cases were classified as a low score, 66 (20.4%) as a middle score, and 37 (11.5%) as a high score in the validation cohort. In a random subset of 100 patients, there was good agreement between the two observers, having a Cohen’s Kappa value of 0.73.

### Association of necrosis score with clinicopathologic characteristics

We further described the characteristics of patients by necrosis score categories in Table 2. In the discovery cohort, in necrosis-high group, tumors were more often located in colon (colon vs. rectum: 68.9% vs. 31.1%, *P* < 0.001), and as compared to stage I, stage III has a higher proportion (stage I vs. III: 2.2% vs. 55.6%, *P* < 0.05). While in necrosis-low group, the proportion of normal CEA was higher (normal vs. abnormal: 81.8% vs. 18.2%, *P* = 0.03). We as well observed similar characteristics trends in the validation cohort of TNM stage (0% vs. 62.2%, *P* < 0.05) and CEA (75.7% vs. 24.3%, *P* = 0.006). There was no significant difference in location, but it was more common in patients with low grade in necrosis-low group (high vs. low: 5.3% vs. 94.7%, *P* = 0.04).

**Table 2:** Baseline characteristics according to necrosis score categories in patients for colorectal cancer in two cohorts

	Necrosis score in discovery cohort (No. %)				Necrosis score in validation cohort (No. %)			
	Low	Middle	High	*P*	Low	Middle	High	*P*
Age								
< 60	155 (72.4)	42 (19.6)	17 (7.9)	0.343	95(63.3)	35(23.3)	20(13.3)	0.228
≥ 60	159 (68.8)	44 (19.0)	28 (12.1)		125(72.3)	31(17.9)	17(9.8)	
Sex								
Male	173 (74.6)	36 (15.5)	23 (9.9)	0.093	118 (65.6)	39 (21.7)	23 (12.8)	0.519
Female	141 (66.2)	50 (23.5)	22 (10.3)		102 (71.3)	27 (18.9)	14 (9.8)	
TNM stage								
I II	64 (88.9)	7 (9.7)	1 (1.4)	0.002	54 (88.5)	7 (11.5)	0 (0.0)	< 0.001
	136 (70.5)	38 (19.7)	19 (9.8)		86 (61.4)	40 (28.6)	14 (10.0)	
III	114 (63.3)	41 (22.8)	25 (13.9)		80 (65.6)	19 (15.6)	23 (18.9)	
Location								
Colon	119 (62.0)	42 (21.9)	31 (16.1)	< 0.001	111 (63.1)	40 (22.7)	25 (14.2)	0.082
Rectum	195 (77.1)	44 (17.4)	14 (5.5)		109 (74.1)	26 (17.7)	12 (8.2)	
CEA level								
Normal	252 (73.9)	59 (17.3)	30 (8.8)	0.026	162 (71.1)	48 (21.1)	18 (7.9)	0.006
Abnormal	56 (60.2)	22 (23.7)	15 (16.1)		52 (59.8)	17 (19.5)	18 (20.7)	
Grade								
Low	277 (71.4)	73 (18.8)	38 (9.8)	0.133	198 (68.8)	60 (20.8)	30 (10.4)	0.042
High	20 (55.6)	10 (27.8)	6 (16.7)		11 (52.4)	4 (19.0)	6 (25.6)	

*P* value was performed by Chi-squared test

*TNM* tumor-node-metastasis, *CEA* carcinoembryonic antigen

### Correlation of T and N categories with necrosis score

**Fig. 2:**
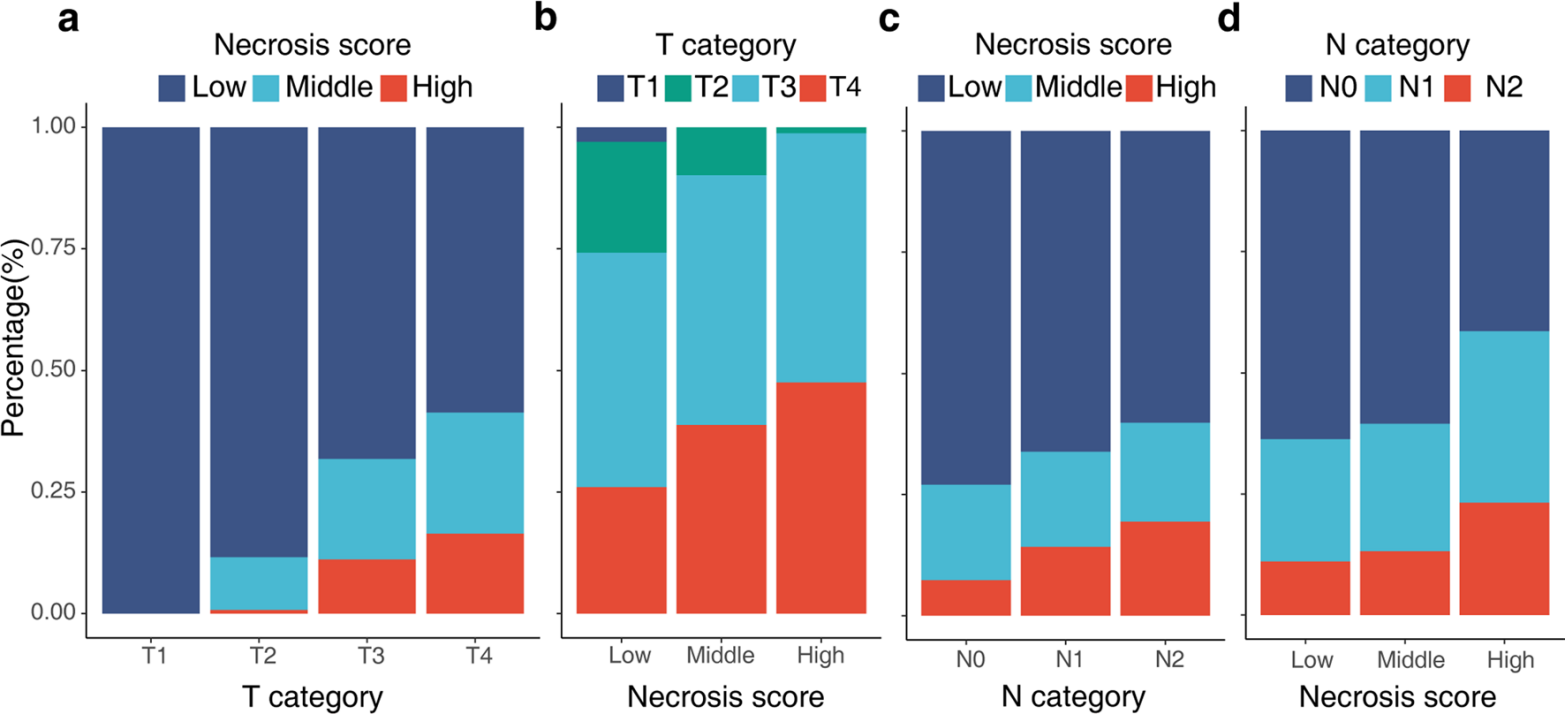
Correlation of T and N categories with necrosis score. **a** The proportion of necrosis-high group increased with the increase of T category (Chi-square test, *P* < 0.0001). **b** The higher the necrosis score, the higher the proportion of high-ranking T category. **c** The proportion of necrosis-high group increased with the increase of N category *(Chi-square test, P* = *0.002)*. **d** The higher the necrosis score, the higher the proportion of high-ranking N category

Only T and N categories were considered. Chi-square test was performed for T and N categories and necrosis score. T and N categories were significantly related to necrosis score. Necrosis-high was associated with a high level of T and N categories (T category, *P* < 0.0001; N category, *P* = 0.002). All T1 (N = 16) and 88.4% of the T2 (N = 138) patients were necrosis-low. In the T3 group (N = 377), this percentage decreased to 68.2%; in the T4 group (N = 237), it was only 58.6%. For the N category, the necrosis-low percentage was 73.0% in the N0 group (N = 466), 66.2% in the N1 group (N = 204) and 60.2% in the N2 group (N = 98) (Fig. 2).

### Prognostic value of necrosis score

**Fig. 3:**
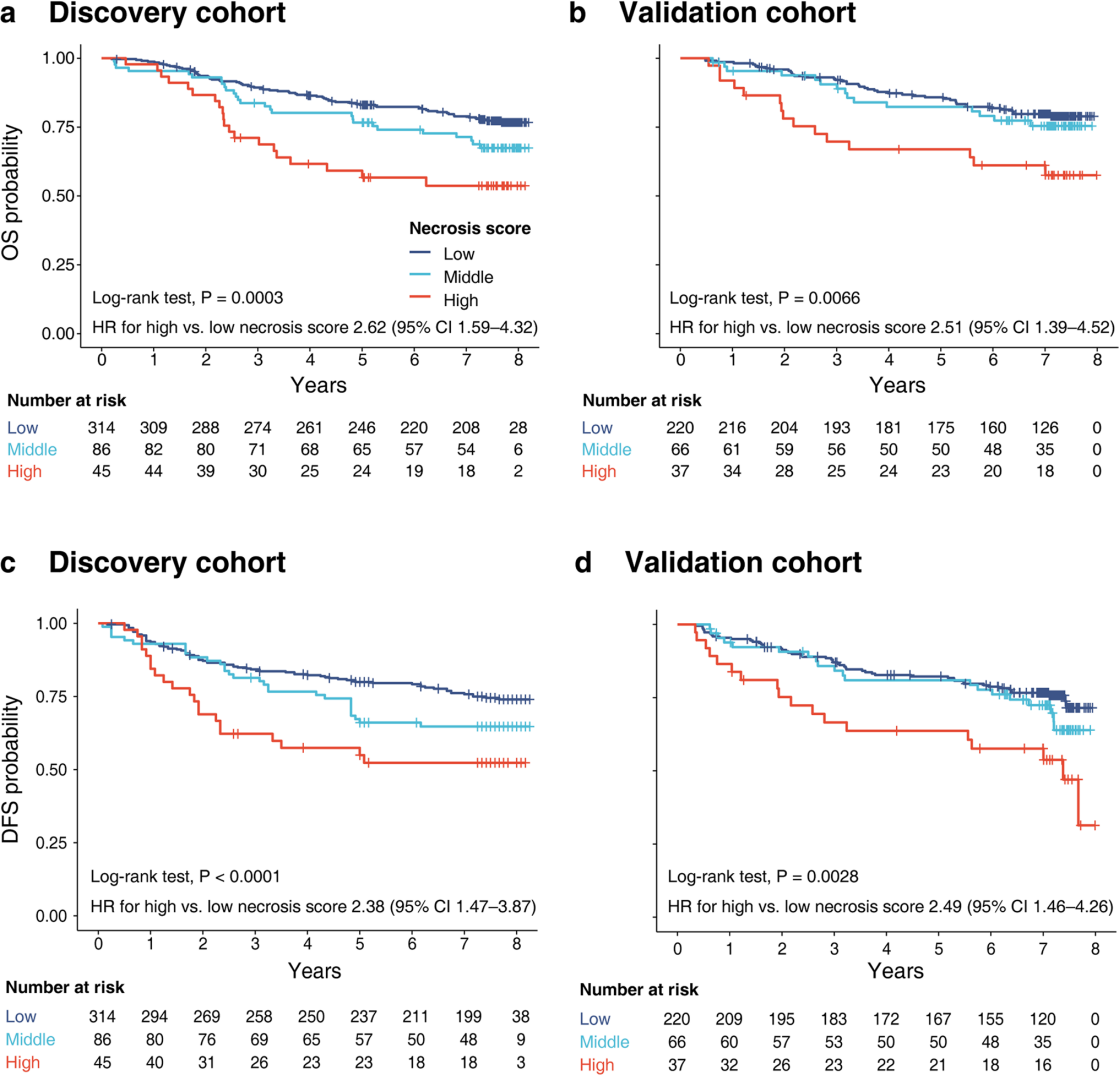
Kaplan–Meier plots for colorectal cancer patients according to necrosis score. Necrosis score for overall survival in the discovery cohort (**a**) and in the validation cohort (**b**). Necrosis score for disease free survival in the discovery cohort (**c**) and in the validation cohort (**d**)

Necrosis-low group was associated with favorable OS (discovery cohort, *P* = 0.0003; validation cohort, *P* = 0.002; Fig. 3a, b). With necrosis score increased, the 5-year OS rates decreased from 89.0% in necrosis-low group to 68.7% in necrosis-high group in discovery cohort. Similar trends were observed in the validation cohort (92.1–69.7%). Unadjusted HR for high vs. low in discovery and validation cohorts was 2.62 (95% CI 1.59–4.32, *P* < 0.0001) and 2.51 (1.39–4.52, *P* = 0.002), respectively (Table 3).

**Table 3:** Uni- and multivariate analyses including TNM stage, sex, age, location, CEA level, grade, and necrosis score for overall survival in two cohorts

	Univariate analysis				Multivariate analysis			
	Discovery cohort		Validation cohort		Discovery cohort		Validation cohort	
	HR (95% CI)	*P*	HR (95% CI)	*P*	HR (95% CI)	*P*	HR (95% CI)	*P*
TNM stage								
I	1		1		1		1	
II	3.71 (1.32–10.4)	0.013	1.81 (0.80–4.14)	0.157	3.50 (1.24–9.87)	0.018	1.71 (0.73–4.02)	0.215
III	10.7 (3.93–29.4)	< 0.001	3.10 (1.38–6.96)	0.006	10.1 (3.66–27.7)	< 0.001	3.35 (1.44–7.79)	0.005
Sex								
Male	1		1					
Female	1.10 (0.76–1.58)	0.622	0.79 (0.49–1.26)	0.320				
Age	1.03 (1.01–1.05)	< 0.001	1.05 (1.03–1.07)	< 0.001	1.03 (1.02–1.05)	< 0.001	1.06 (1.04–1.09)	< 0.001
Location								
Colon	1		1					
Rectum	1.03 (0.71–1.49)	0.881	1.44 (0.91–2.28)	0.120				
CEA level^a^								
Normal	1		1				1	
Abnormal	1.45 (0.96–2.19)	0.077	2.00 (1.24–3.24)	0.005			1.68 (1.02–2.78)	0.043
Grade^a^								
Low	1		1					
High	1.87 (1.08–3.21)	0.024	1.67 (0.76–3.65)	0.198				
Necrosis score								
Low	1		1		1		1	
Middle	1.50 (0.96–2.34)	0.076	1.21 (0.67–2.18)	0.520	1.23 (0.79–1.93)	0.360	1.41 (0.76–2.64)	0.276
High	2.62 (1.59–4.32)	< 0.001	2.51 (1.39–4.52)	0.002	1.86 (1.12–3.08)	0.016	2.09 (1.12–3.91)	0.021

*TNM* tumor-node-metastasis, *CEA* carcinoembryonic antigen, *HR* hazard ratio, *CI* confidence interval

^a^Multivariate analysis was performed only on patients with complete data (n = 315)

Obtained results showed that necrosis score was significantly correlated with DFS, with unadjusted HR for high vs. low being 2.38 (95% CI 1.47–3.87, *P* < 0.0001) in discovery cohort and 2.49 (1.46–4.26, *P* = 0.001) in validation cohort (Supplementary Table 2). The 3-year DFS rates declined with necrosis score increased. In necrosis-low group, the 3-year DFS rates was 83.6%, decreased to 59.8% in necrosis-high group (*P* < 0.0001; Fig. 3c). In the validation cohort, these findings were confirmed. In validation cohort, they were 86.5% in necrosis-low group, compared with 66.5% in high group (*P* = 0.003; Fig. 3d).

### Survival analysis of necrosis score stratified with clinicopathological characteristics

In addition, we examined whether necrosis score could be applied to subgroups of patients with clinicopathological characteristics. In DFS and OS, based on stratification by age (≥ 60), sex (female and male), grade-low, location (colon and rectum), and stage III, the score still had a statistically significant impact on the prognosis. (All *P* < 0.05; Supplementary Figs. S1–S5). The score had no statistically significant impact on the prognosis stratified by the other clinicopathological characteristics (All *P* > 0.05).

### Necrosis score as an independent prognostic factor for OS and DFS

We performed univariate analyses on age, sex, TNM stage, location, CEA level, tumor grade, and necrosis score. In univariate Cox analyses, characteristics that reached significance for OS were age, grade, TNM stage, and necrosis score (Table 3, all *P* < 0.05). In the multivariate analysis, necrosis score was an independent prognostic marker for improved OS (discovery cohort: adjusted HR for high vs. low 1.86, 95% CI 1.12–3.08, *P* = 0.02; validation cohort: 2.09, 1.12–3.91, 0.02, Table 3). We identified age, grade, CEA level, TNM stage, and necrosis score as independent predictors for DFS (Supplementary Table 2, all *P* < 0.05). In multivariate analysis, necrosis score was still associated with DFS, independent of age, CEA level, grade, and TNM stage (discovery cohort: adjusted HR for high vs. low 1.71, 95% CI 1.05–2.79, *P* = 0.03; validation cohort: 2.00, 1.13–3.53, 0.02, Supplementary Table 2).

To evaluate the added prognostic value of necrosis score, we developed three Cox models: stage, necrosis score, and combined them (Table 4). In the discovery cohort, the C-index for necrosis score to predict OS was 0.578 (95% CI 0.532–0.625). The addition of TNM stage to necrosis score increased for predicting the OS from 0.578 to 0.706. Similar to the validation cohort, the C-index increases to 0.627 (0.561–0.692) after combining the above two. The C-index for DFS of necrosis score was 0.572 (0.528–0.616) and raised to 0.705 (0.666–0.744) by adding stage in the discovery cohort. Similarly, the C-index for DFS was 0.565 (0.510–0.620) when including only necrosis score and 0.628 (0.568–0.688) when adding stage in the validation cohort.

**Table 4:** The discrimination performance of necrosis score and TNM stage for predicting OS and DFS in two cohorts

		Discovery cohort	Validation cohort
		C-index (95% CI)	C-index (95% CI)
Stage	DFS	0.689 (0.652–0.726)	0.609 (0.554–0.664)
	OS	0.686 (0.647–0.725)	0.605 (0.545–0.664)
Necrosis score	DFS	0.572 (0.528–0.616)	0.565 (0.510–0.620)
	OS	0.578 (0.532–0.625)	0.572 (0.512–0.633)
Stage + necrosis score	DFS	0.705 (0.666–0.744)	0.628 (0.568–0.688)
	OS	0.706 (0.664–0.748)	0.627 (0.561–0.692)

*TNM* tumor-node-metastasis, *OS* overall survival, *DFS* disease free survival, *CI* confidence interval

Starting from the logistic model containing all prognostic factors, we removed some statistically insignificant factors (*P* > 0.05) and kept the resulting model as the basis for the nomogram. In the discovery cohort, nomograms incorporating the respective independent prognostic factors of OS and DFS were established (Supplementary Fig. S6).

### Survival analysis of necrosis score stratified with MSI status

We further examined that based on stratification by MSI, the score had a statistically significant impact on the prognosis for OS (*P* = 0.005) and DFS (*P* = 0.02) (Supplementary Fig. S7). As necrosis score, only a marginally statistically significant was found among microsatellite instability (MSS) individuals in DFS (*P* = 0.06; Supplementary Fig. S7d), while the difference was no longer presented in OS (*P* = 0.15; Supplementary Fig. S7c).

### Association of necrosis score with adjuvant chemotherapy in stage II CRC

**Fig. 4:**
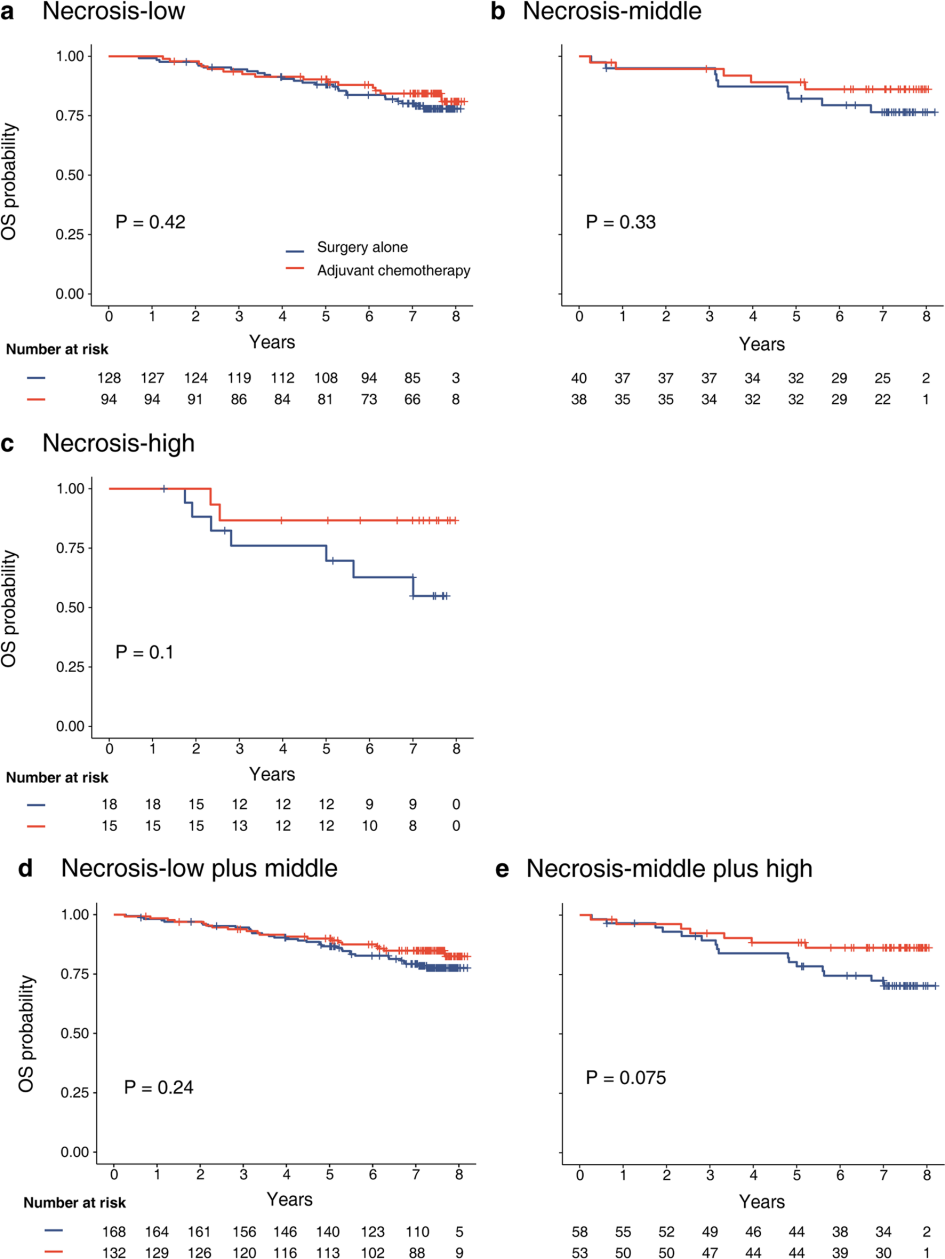
Kaplan–Meier plots of effect of adjuvant chemotherapy on overall survival in different subgroups in stage II colorectal cancer. **a** Necrosis-low group; **b** Necrosis-middle group; **c** Necrosis-high group; **d** Necrosis-low plus middle group; **e** Necrosis-middle plus high group. *OS* overall survival

In stage II CRC, the OS and DFS were stratified, accounting for necrosis score, including necrosis-low, middle, high, necrosis-low plus middle and necrosis-middle plus high groups. The obtained findings exhibited that in necrosis-middle plus high group, there was a marginal difference in OS between surgery alone group and adjuvant chemotherapy group (78.4% vs. 86.3%, *P* = 0.80; Fig. 4e), while the difference was no longer present in DFS (78.4% vs. 80.2%, *P* = 0.32; Supplementary Fig. S8e). In stratified to necrosis-low, middle, high, and necrosis-low plus middle groups, OS and DFS had no difference between the two groups (all *P* > 0.05, OS: Fig. 4a–d; DFS: Supplementary Fig. S8a–d).

## Discussion and conclusion

In this study, we found that the necrosis score was a stable prognosis factor in two independent cohorts of CRC, independent of TNM stage and other clinicopathological factors, and had the potential ability to predict the curative effect of adjuvant chemotherapy in stage II CRC.

Necrosis has been receiving much attention in the prognosis prediction of CRC [14, 20, 21]. Increased hypoxia due to abnormal rates of cell division, development of solid tumor masses that disrupt vascular penetration, resulting in decreased oxygen diffusion [22]. Tumor necrosis results from the inability to meet the requirement for the rapid proliferation of tumor cells [5, 6]. Necrosis was the final outcome of various pathophysiological processes after the body adapted and regulated. During the avascular phase, tumor mainly relies on the diffusion of surrounding tissues to acquire nutrients and excrete metabolites [23], thereby limiting its growth. During the vascular phase, angiogenesis and production of angiogenic factors were fundamental for tumor progression in form of growth, invasion, and metastasis [24]. New vessels adapted to hypoxia caused by rapid proliferation by conveying oxygen and nutrients. However, blood flow in many tumors is disorganized and variable [25]. First, the arteriovenous pressure difference decreases, and the viscous and geometric resistance increases in tumor, which will exert a compressive effect on blood vessels [26], which will increase blood flow resistance and damage the tumor blood supply. Secondary, compared with normal tissues, functional lymphatic capillaries were absent from the interior of solid tumors [27], which leads to increased interstitial fluid pressure within them [28]. Increased interstitial fluid pressure inhibits the distribution of larger molecules through convection [29] and compresses blood vessels, causing blood to shift from the tumor center to the periphery, resulting in decreased blood supply to the tumor center. These indicated that even though neovascularization could weaken the effects of hypoxia, there were various factors that could affect neovascularization. High tumor necrosis means that even after adaptation and regulation, the body still could not reverse the effect of hypoxia, suggesting worse prognosis. The above explained the results of our work. A high proportion of necrosis corresponded to the low DFS and OS in the two cohorts. The 5-year OS rate of patients in necrosis-high group was much lower than that in necrosis-low group (68.7% vs. 89.0%), consistent with previous studies [14]. The 3-year DFS rate was recorded for 83.6% of patients with a low score and 59.8% with high. The results showed that necrosis score achieved comparable prognostic performance. Multivariate analysis also further confirmed. Validation of necrosis score quantified in HE-stained WSIs by two independent cohorts can support the value of necrosis score for stratifying the risk of CRC patients.

TNM stage remained one of the most clinically applied prognostic factors in CRC, including the depth of local invasion into the bowel wall and the infiltration of regional lymph nodes [30, 31]. As expected, the percentage of necrosis-high group grows with the increase in T and N categories. T category increased with the increase of tumor volume and the extent of adjacent tissue involvement. The larger the tumor volume or the more profound the invasion, the upper the T category and the more prominent tumor necrosis [32]. Additionally, as the tumor grows, the body is uncompensated for the tumor’s blood supply requirement, and necrosis occurs [5, 6]. In other words, with the increase of tumor size or infiltration, the blood supply could not meet the needs of tumor growth, and the chance of tumor necrosis would increase. As shown in the results, with the increase in T category, the proportion of necrosis-low group decreased, with 88.4% of the T2 patients were necrosis-low group. In the T4 group, this percentage decreased to only 58.6%. Lymph nodes metastasis indicated that the tumor has strong metastatic ability and a great degree of aggressiveness, suggesting a poor prognosis for the patient [33]. Tumor cells break away from the primary tumor, invade the basement membrane [34], infiltrate and grow in the surrounding stroma and involve the lymphatic vessels, survive in the lymphatic vessels, and are metastasized to the lymph nodes to form metastatic tumors [35, 36]. In our result, the proportion of positive lymph nodes (N^+^) increased with the increase of necrosis score. The great extent of necrosis indicated the rapid proliferation of tumor cells and the powerful metastatic potential, suggesting that necrosis score has the potential to speculate on regional lymph node metastasis.

The stage of CRC was assigned according to TNM stage system of UICC, which provided treatment guidelines. Consensus has been reached for the treatment of stage I CRC with surgery alone and chemotherapy in addition to surgery for stage III/IV CRC, but the overall benefit of adjuvant chemotherapy after resection for stage II CRC remains unclear [37]. MSI was not only an independent prognostic factor for stage II CRC [38] but also a predictor of the efficacy of adjuvant chemotherapy. According to the latest ESMO and CSCO guidelines, adjuvant chemotherapy was not recommended, but observation and follow-up for patients with stage II CRC without high-risk factors such as MSI-H [16, 39]. For stage II MSI-H CRC patients, current studies found that patients treated with 5-FU single-agent adjuvant chemotherapy had no survival benefit [40, 41]. However, in the MSI-H stratified analysis, it was found that there were significant differences in OS (*P* = 0.005) and DFS (*P* = 0.02) with different necrosis scores. Patients with necrosis-high had a lower survival rate than those with necrosis-low, suggesting that necrosis in patients with MSI-H could further risk stratified, and the treatment of the stage II population still needed to be stratified and analyzed to achieve a more individualized treatment. On the other hand, we also observed that in patients with stage II CRC, in necrosis-middle plus high group, it was found that there was a trend but no significant difference between surgery alone group and adjuvant chemotherapy group (78.4% vs. 86.3%, *P* = 0.075). It was speculated that adjuvant chemotherapy could improve the survival rate with a high proportion of necrosis. Therefore, according to necrosis score, we speculate that patients with stage II MSI-H CRC could be further selected suitably for adjuvant chemotherapy. Further studies are needed to verify whether patients with high necrosis score are a high-risk factor for adjuvant chemotherapy in stage II MSI-H CRC to provide more accurate and individualized treatment for patients in the future.

One of the limitations of our study is that this analysis is retrospective and may be susceptible to bias introduced by certain risk factors. Therefore, it is necessary to conduct prospective studies to verify the effectiveness of necrosis score in routine clinical applications. Secondly, in this study, WSIs was used to ameliorate differences in reporting among pathologists and the difficulty of scaling up the evaluation process under a microscope. But in our study, necrosis score was still a not full-quantified method that was inconvenient to popularize widely in daily work. Therefore, using image segmentation to quantify tumor necrosis automatically is one of our future research ambitions.

In conclusion, we used a necrosis score for semi-quantitative using HE-stained WSI in CRC. We found evidence that necrosis score has a stable prognostic value in CRC, with a higher necrosis score being more unfavorable for overall survival. Necrosis score might provide vital assistance for changes in risk stratification in stage II CRC patients to perform adjuvant chemotherapy.

## Supplementary Information

Supplementary file1: (PDF 1799 KB)

## Data Availability

The datasets used and/or analyzed during the current study are available from the corresponding author on reasonable request.
