# Supplementary material for: Necrosis score as a prognostic factor in stage I–III colorectal cancer: a retrospective multicenter study
Source: Discov Oncol. 2023 May 8;14:61. doi: 10.1007/s12672-023-00655-w (PMC10167085; doi:10.1007/s12672-023-00655-w)
Supplement: Supplementary file 1 — Supplementary file1: (PDF 1799 KB) [file 12672_2023_655_MOESM1_ESM.pdf]

Additional file 1

Necrosis score as a prognostic factor in stage I–III colorectal cancer: A retrospective multicenter study

Journal's name: Discover Oncology

Authors: Huifen Ye[1,2,3#], Yiting Wang[4#], Su Yao[5#], Zaiyi Liu[1,2,3], Changhong Liang[1,2,3], Yaxi Zhu[4*], Yanfen Cui[6*], Ke Zhao[1,3,7*]

Affiliations:

[1] Department of Radiology, Guangdong Provincial People's Hospital (Guangdong Academy of Medical Sciences),Southern Medical University, Guangzhou, China

[2] The Second School of Clinical Medicine, Southern Medical University, Guangzhou, China

[3] Guangdong Provincial Key Laboratory of Artificial Intelligence in Medical Image Analysis and Application, Guangzhou, China

[4] Department of Pathology, The Sixth Affiliated Hospital of Sun Yat-sen University, Guangzhou, China

[5] Department of Pathology, Guangdong Provincial People's Hospital (Guangdong Academy of Medical Sciences),Southern Medical University, Guangzhou, China

[6] Department of Radiology, Shanxi Cancer Hospital, Shanxi Medical University, Taiyuan, China

[7] Guangdong Cardiovascular Institute, Guangdong Provincial People's Hospital, Guangdong Academy of Medical Sciences, Guangzhou, China

# These authors contributed equally to this work.

[*]**Corresponding authors:**

Yaxi Zhu, zhuyax@mail.sysu.edu.cn

Address: Department of Pathology, The Sixth Affiliated Hospital of Sun Yat-Sen University, 26 Yuan Cun 2 Cross Road, TianHe District, Guangzhou, 510655, China; Tel&Fax: 0086-2083870125

Or Yanfen Cui, yanfen210@126.com

Address: Department of Radiology, Shanxi Cancer Hospital, Shanxi Medical University, No.3, Xinjie West Alley, Taiyuan, 030013, China; Tel&Fax: 0086-2083870125

Or Ke Zhao, zhaoke@gdph.org.cn

Address: Guangdong Provincial Key Laboratory of Artificial Intelligence in Medical Image Analysis and Application, Guangdong Provincial People's Hospital, Guangzhou, China, 106 Zhongshan Er Road, Guangzhou, 510080, China; Tel&Fax: 0086-2083870125

This PDF file includes:

Figure (S1–S8)

Table (S1-2)

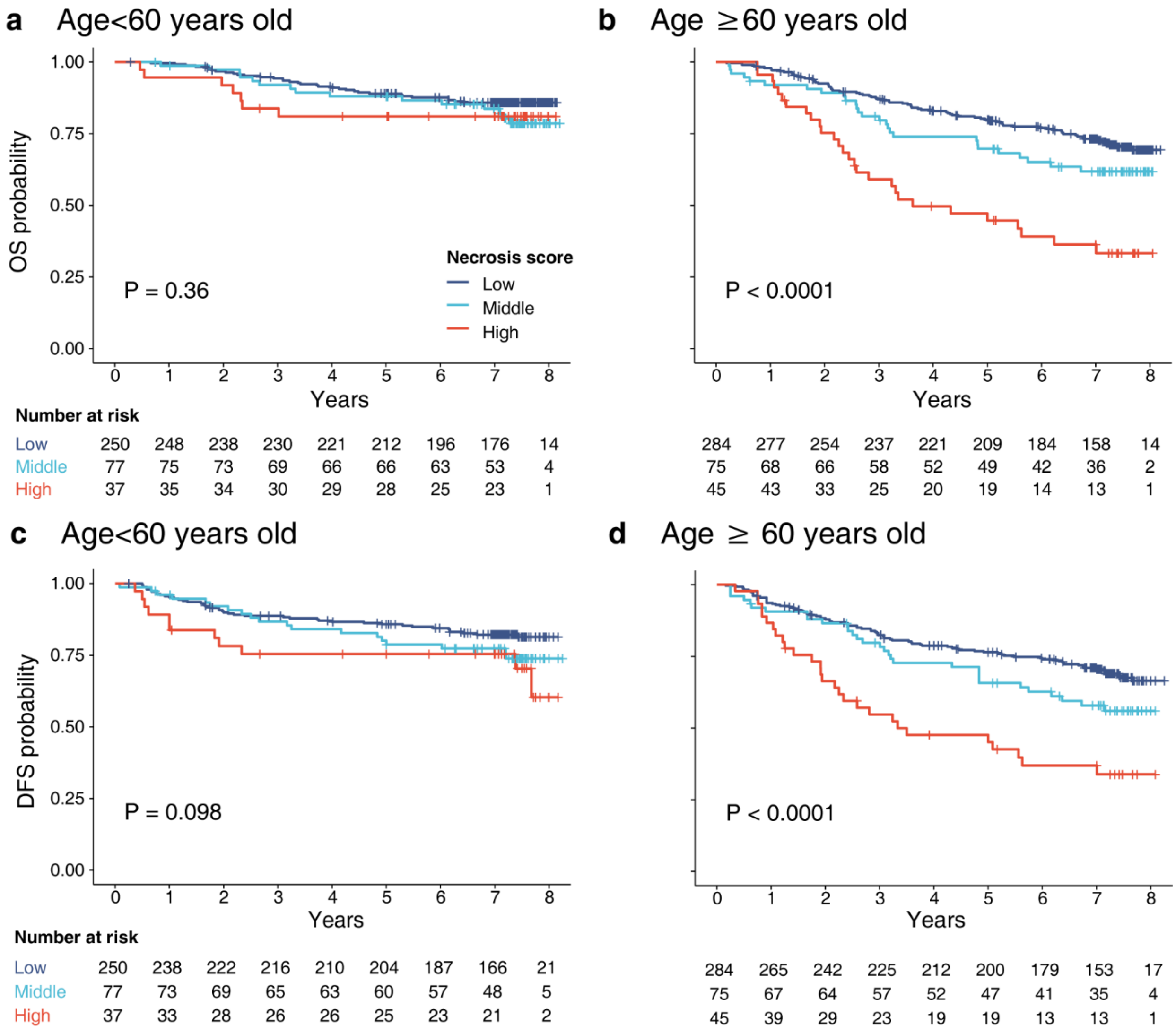

Fig. S1 Kaplan–Meier plots of estimated OS (a-b) and DFS (c-d) for all patients according to necrosis score, stratified by age OS, overall survival; DFS, disease free survival.

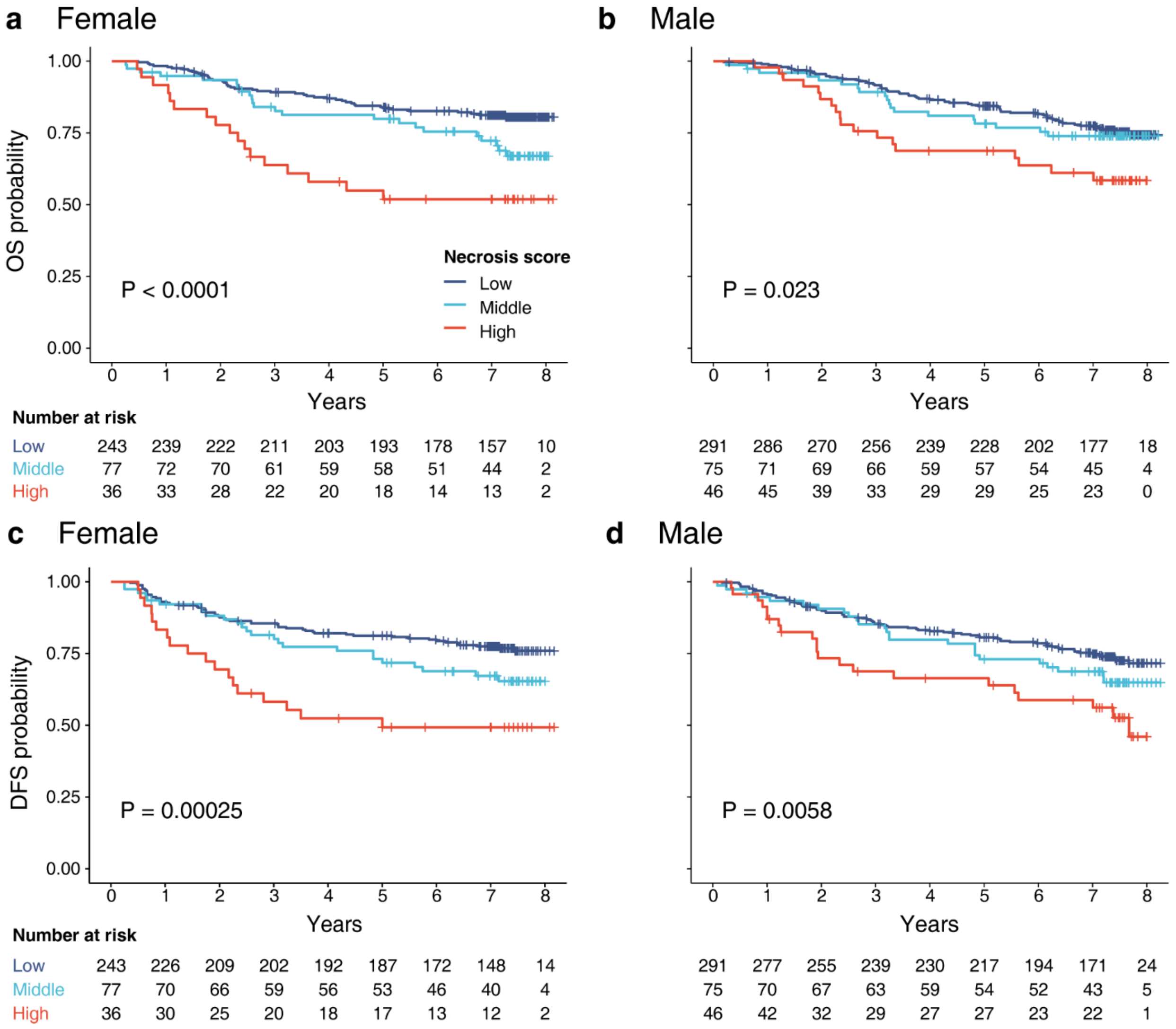

Fig. S2 Kaplan–Meier plots of estimated OS (a-b) and DFS (c-d) for all patients according to necrosis score, stratified by sex OS, overall survival; DFS, disease free survival.

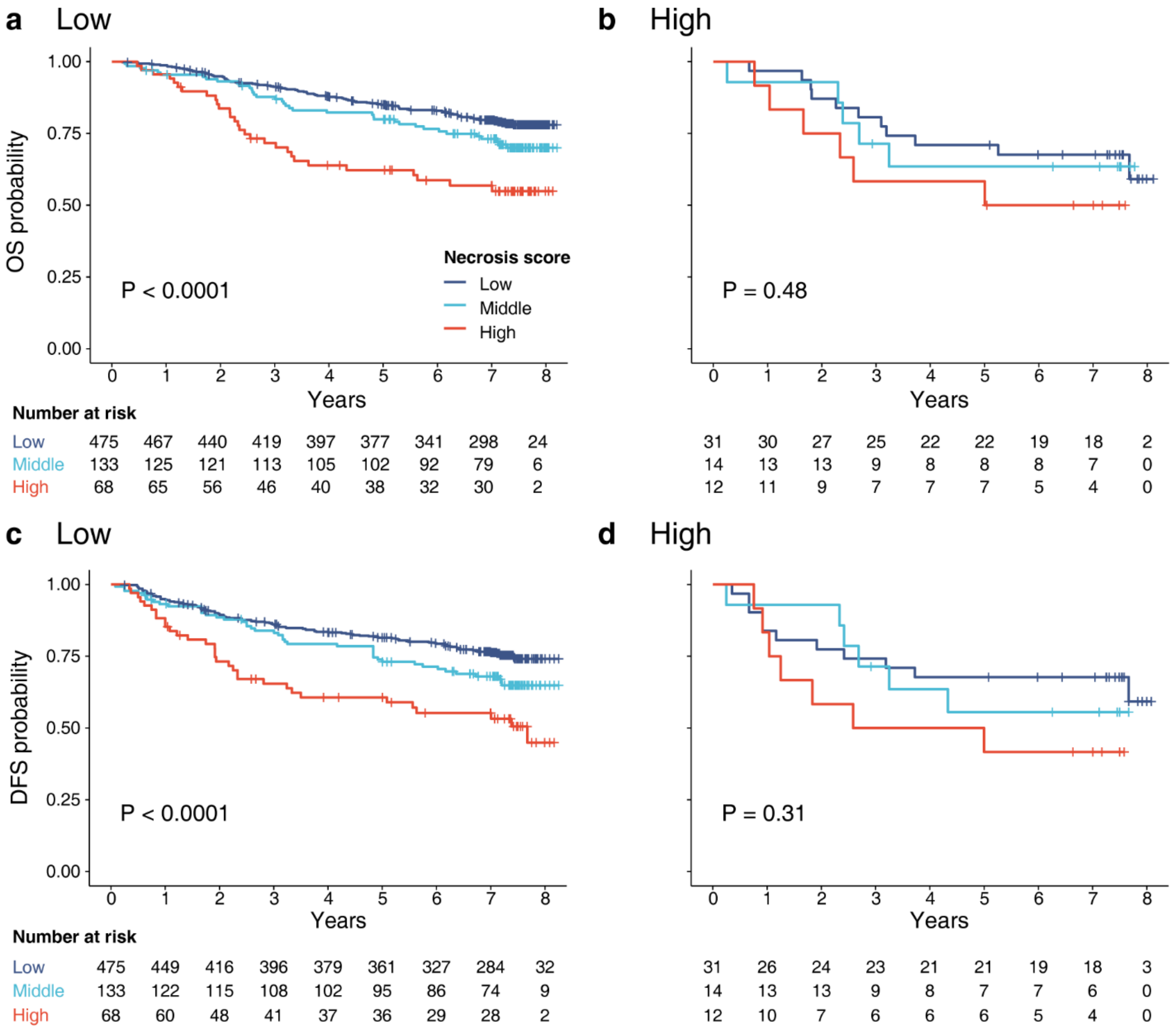

Fig. S3 Kaplan–Meier plots of estimated OS (a-b) and DFS (c-d) for all patients according to necrosis score, stratified by grade OS, overall survival; DFS, disease free survival.

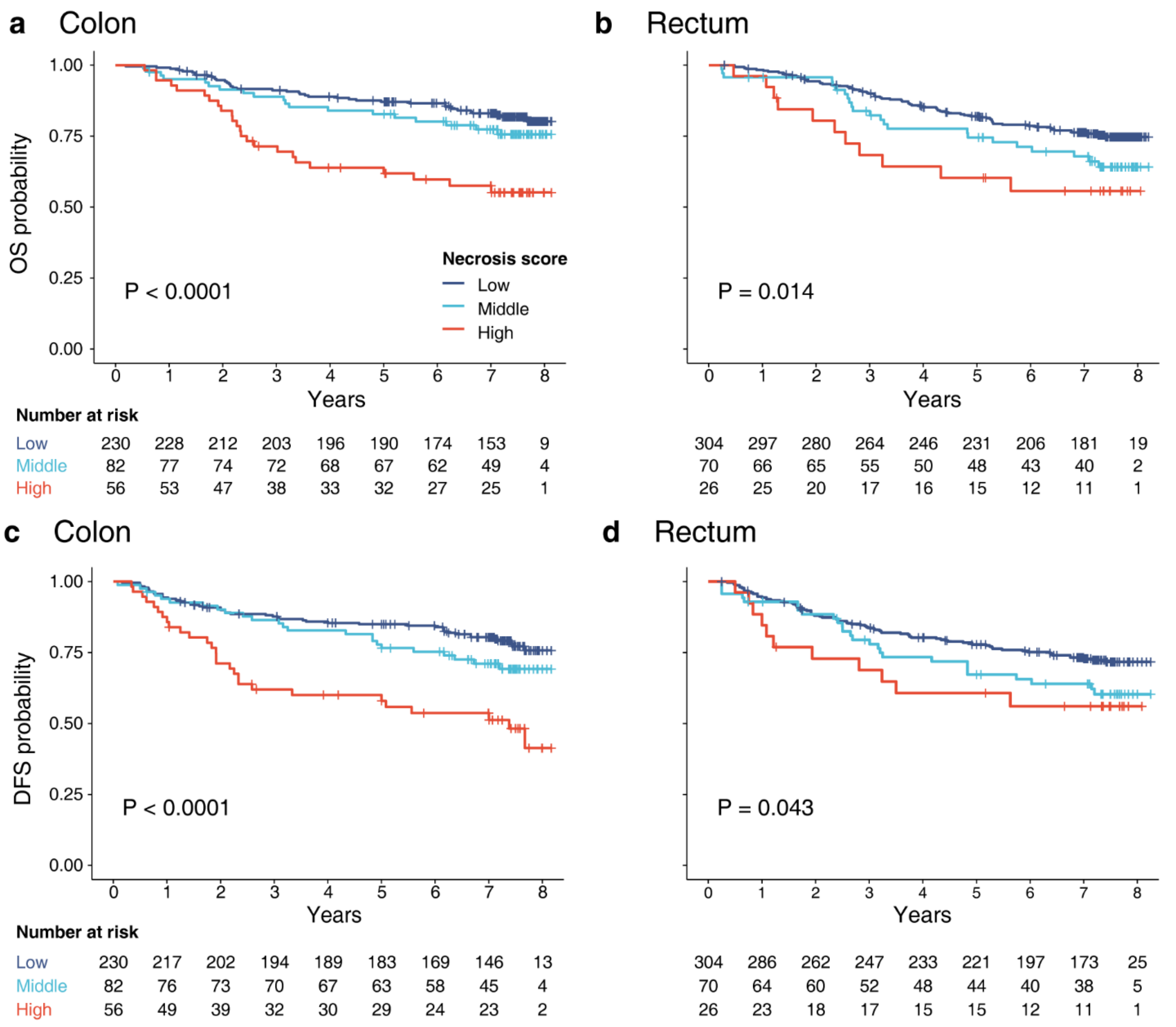

Fig. S4 Kaplan–Meier plots of estimated OS (a-b) and DFS (c-d) for all patients according to necrosis score, stratified by location OS, overall survival; DFS, disease free survival.

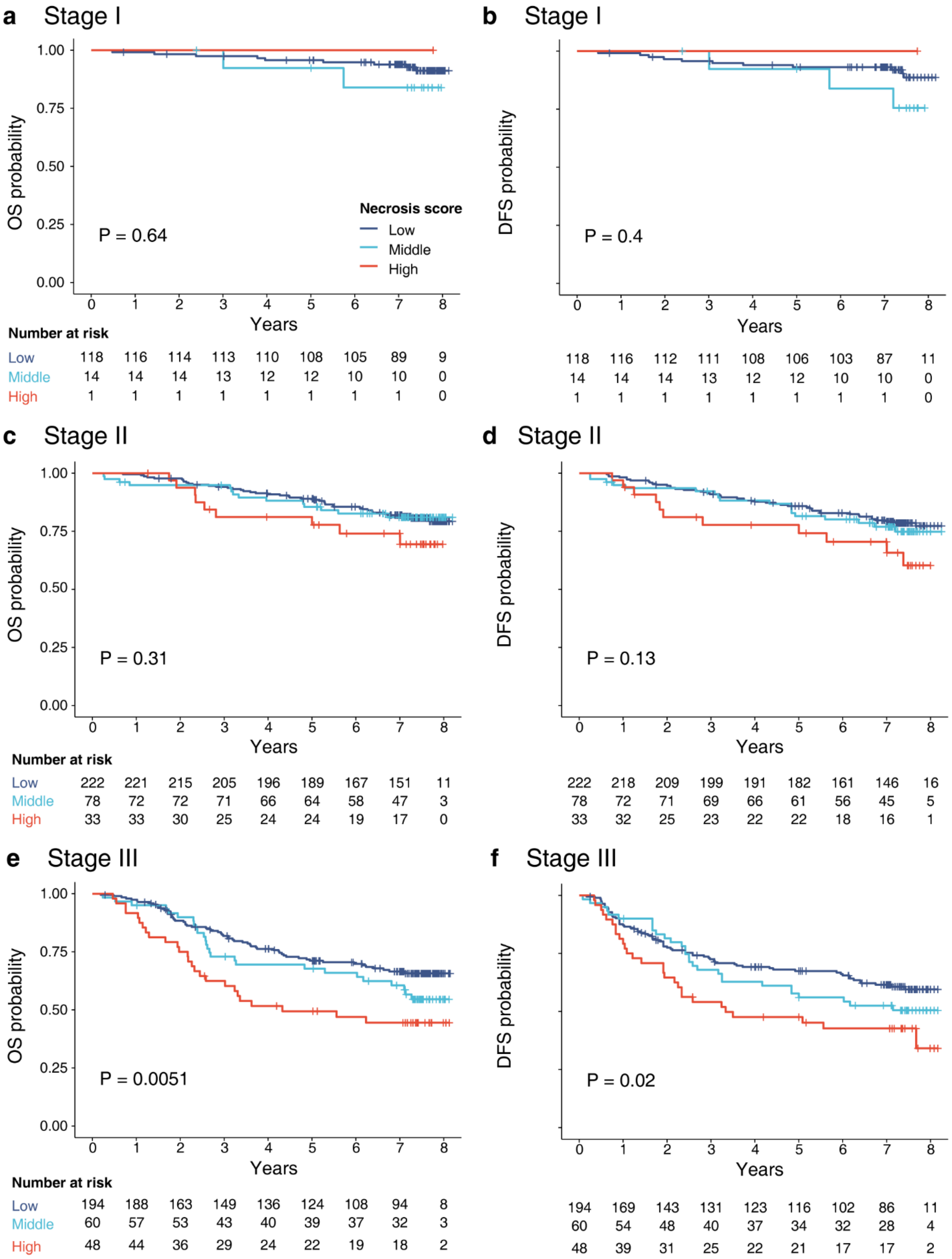
a
Stage I
OS probability
1.00
0.75
0.50
0.25
0.00
P = 0.64
Necrosis score
Low
Middle
High
0 1 2 3 4 5 6 7 8
Years
Number at risk
Low 118 116 114 113 110 108 105 89 9
Middle 14 14 14 13 12 12 10 10 0
High 1 1 1 1 1 1 1 1 0
b
Stage I
DFS probability
P = 0.4
0 1 2 3 4 5 6 7 8
Years
118 116 112 111 108 106 103 87 11
14 14 14 13 12 12 10 10 0
1 1 1 1 1 1 1 1 0
c
Stage II
OS probability
1.00
0.75
0.50
0.25
0.00
P = 0.31
0 1 2 3 4 5 6 7 8
Years
Number at risk
Low 222 221 215 205 196 189 167 151 11
Middle 78 72 72 71 66 64 58 47 3
High 33 33 30 25 24 24 19 17 0
d
Stage II
DFS probability
P = 0.13
0 1 2 3 4 5 6 7 8
Years
222 218 209 199 191 182 161 146 16
78 72 71 69 66 61 56 45 5
33 32 25 23 22 22 18 16 1
e
Stage III
OS probability
1.00
0.75
0.50
0.25
0.00
P = 0.0051
0 1 2 3 4 5 6 7 8
Years
Number at risk
Low 194 188 163 149 136 124 108 94 8
Middle 60 57 53 43 40 39 37 32 3
High 48 44 36 29 24 22 19 18 2
f
Stage III
DFS probability
P = 0.02
0 1 2 3 4 5 6 7 8
Years
194 169 143 131 123 116 102 86 11
60 54 48 40 37 34 32 28 4
48 39 31 25 22 21 17 17 2

Fig. S5 Kaplan–Meier plots of estimated OS (a, c, e) and DFS (b, d, f) for all patients according to necrosis score, stratified by TNM stage OS, overall survival; DFS, disease free survival; TNM, tumor-node-metastasis.

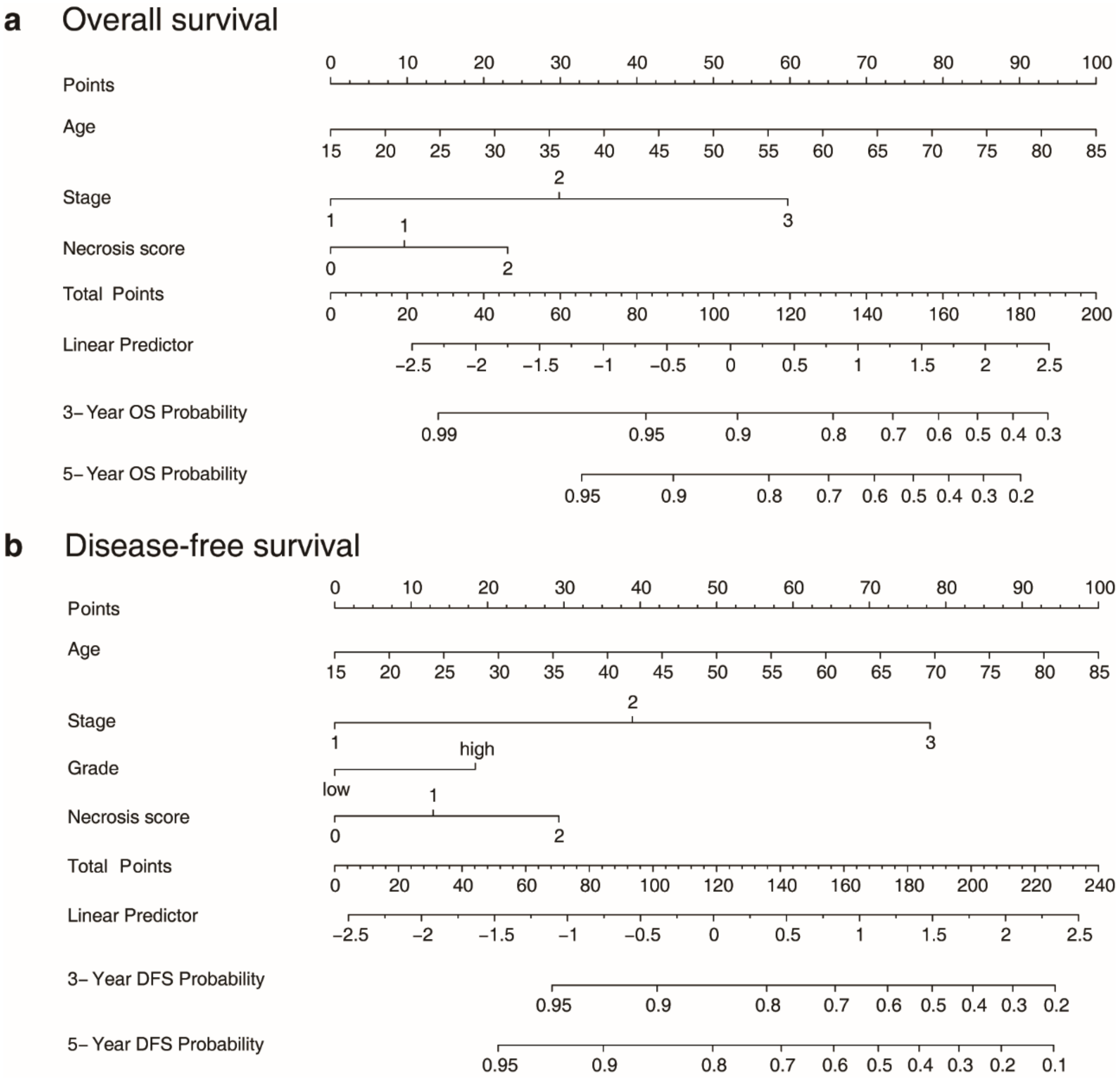

Fig. S6 Colorectal cancer survival nomogram. Nomograms for predicting overall survival (a) and (b) disease-free survival. OS, overall survival; DFS, disease-free survival.

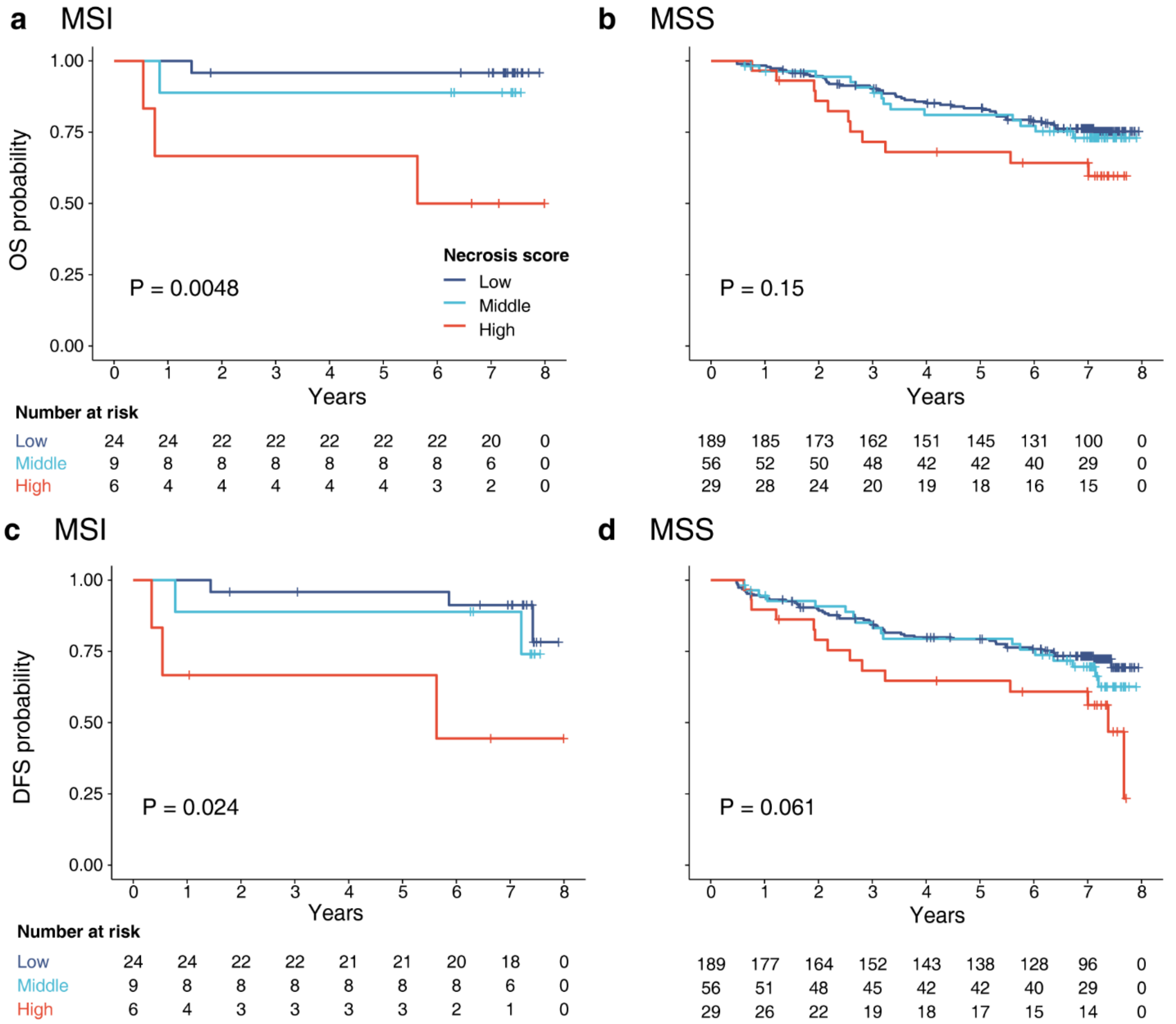

Fig. S7 Kaplan–Meier plots of estimated OS (a-b) and DFS (c-d) for all patients according to necrosis score, stratified by MSI status MSI, microsatellite instability; MMS, microsatellite stability; OS, overall survival; DFS, disease free survival.

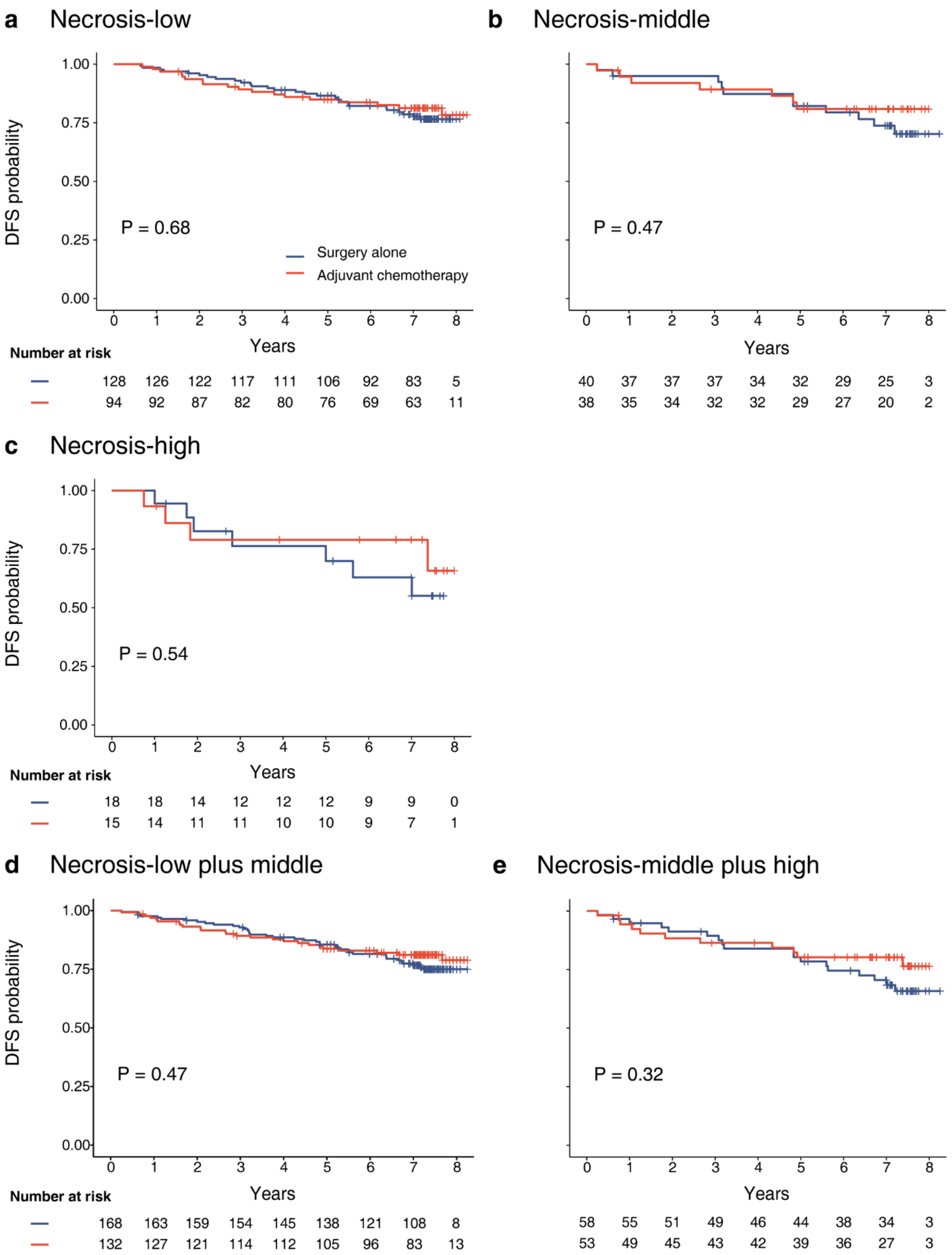
a Necrosis-low
DFS probability
1.00
0.75
0.50
0.25
0.00
P = 0.68
Surgery alone
Adjuvant chemotherapy
0 1 2 3 4 5 6 7 8
Years
Number at risk
128 126 122 117 111 106 92 83 5
94 92 87 82 80 76 69 63 11
b Necrosis-middle
P = 0.47
0 1 2 3 4 5 6 7 8
Years
40 37 37 37 34 32 29 25 3
38 35 34 32 32 29 27 20 2
c Necrosis-high
DFS probability
1.00
0.75
0.50
0.25
0.00
P = 0.54
0 1 2 3 4 5 6 7 8
Years
Number at risk
18 18 14 12 12 12 9 9 0
15 14 11 11 10 10 9 7 1
d Necrosis-low plus middle
DFS probability
1.00
0.75
0.50
0.25
0.00
P = 0.47
0 1 2 3 4 5 6 7 8
Years
Number at risk
168 163 159 154 145 138 121 108 8
132 127 121 114 112 105 96 83 13
e Necrosis-middle plus high
P = 0.32
0 1 2 3 4 5 6 7 8
Years
58 55 51 49 46 44 38 34 3
53 49 45 43 42 39 36 27 3

Fig. S8 Kaplan–Meier plots of effect of adjuvant chemotherapy on disease free survival in different subgroups in stage II colorectal cancer (a) Necrosis-low group; (b) Necrosis-middle group; (c) Necrosis-high group; (d) Necrosis-low plus middle group; (e) Necrosis-middle plus high group. DFS, disease free survival.

Table S1. The distributions of post-surgery treatment of stage II colorectal cancer patients

	**Surgery alone**	**Adjuvant chemotherapy**
**Stage II**	186 (55.9%)	147 (44.1%)

Table S2. Uni– and multivariate analyses including TNM stage, sex, age, location, CEA level, grade, and necrosis score for DFS in the two cohort.

	Univariate analysis				Multivariate analysis			
	Discovery cohort		Validation cohort		Discovery cohort		Validation cohort	
	**HR (95% CI)**	***P***	**HR (95% CI)**	***P***	**HR (95% CI)**	***P***	**HR (95% CI)**	***P***
**TNM stage**								
I	1		1		1		1	
II	4.33 (1.55–12.2)	0.005	1.50 (0.74–3.03)	0.262	3.82 (1.36–10.7)	0.011	1.43 (0.69–2.97)	0.335
III	12.2 (4.45–33.2)	<0.001	2.70 (1.36–5.36)	0.004	10.2 (3.71–28.1)	<0.001	2.84 (1.37–5.86)	0.005
**Sex**								
Male	1		1					
Female	1.09 (0.77–1.55)	0. 613	0.81 (0.53–1.24)	0.337				
**Age**	1.02 (1.00–1.03)	0.019	1.04 (1.02–1.06)	<0.001	1.02 (1.01–1.04)	0.007	1.05 (1.03–1.07)	<0.001
**Location**								
Colon	1		1					
Rectum	0.99 (0.70–1.41)	0.966	1.30 (0.86–1.96)	0.217				
**CEA level***								
Normal	1		1				1	
Abnormal	1.47 (0.99–2.18)	0.055	2.05 (1.33–3.16)	0.001			1.62 (1.04–2.54)	0.034
**Grade****								
High	1		1		1			
Low	1.97 (1.18–3.28)	0.009	1.28 (0.59–2.77)	0.532	1.74 (1.04–2.93)	0.035		
**Necrosis score**								
Low	1		1		1		1	
Middle	1.48 (0.97–2.25)	0.070	1.28 (0.77–2.15)	0.341	1.17 (0.76–1.79)	0.471	1.48 (0.86–2.55)	0.154
High	2.38 (1.47–3.87)	<0.001	2.49 (1.46–4.26)	0.001	1.71 (1.05–2.79)	0.032	2.00 (1.13–3.53)	0.017

Note: *Multivariate analysis was performed only on patients with complete data (n = 315). **Multivariate analysis was performed only on patients with complete data (n = 424).

Abbreviations: TNM, tumor-node-metastasis; CEA, carcinoembryonic antigen; DFS, disease free survival; HR, hazard ratio; CI, confidence interval.
